# Supplementary figures and images for: Managing uncertainty in metabolic network structure and improving predictions using EnsembleFBA
Source: PLoS Comput Biol. 2017 Mar 6;13(3):e1005413. doi: 10.1371/journal.pcbi.1005413 (PMC5358886; doi:10.1371/journal.pcbi.1005413)

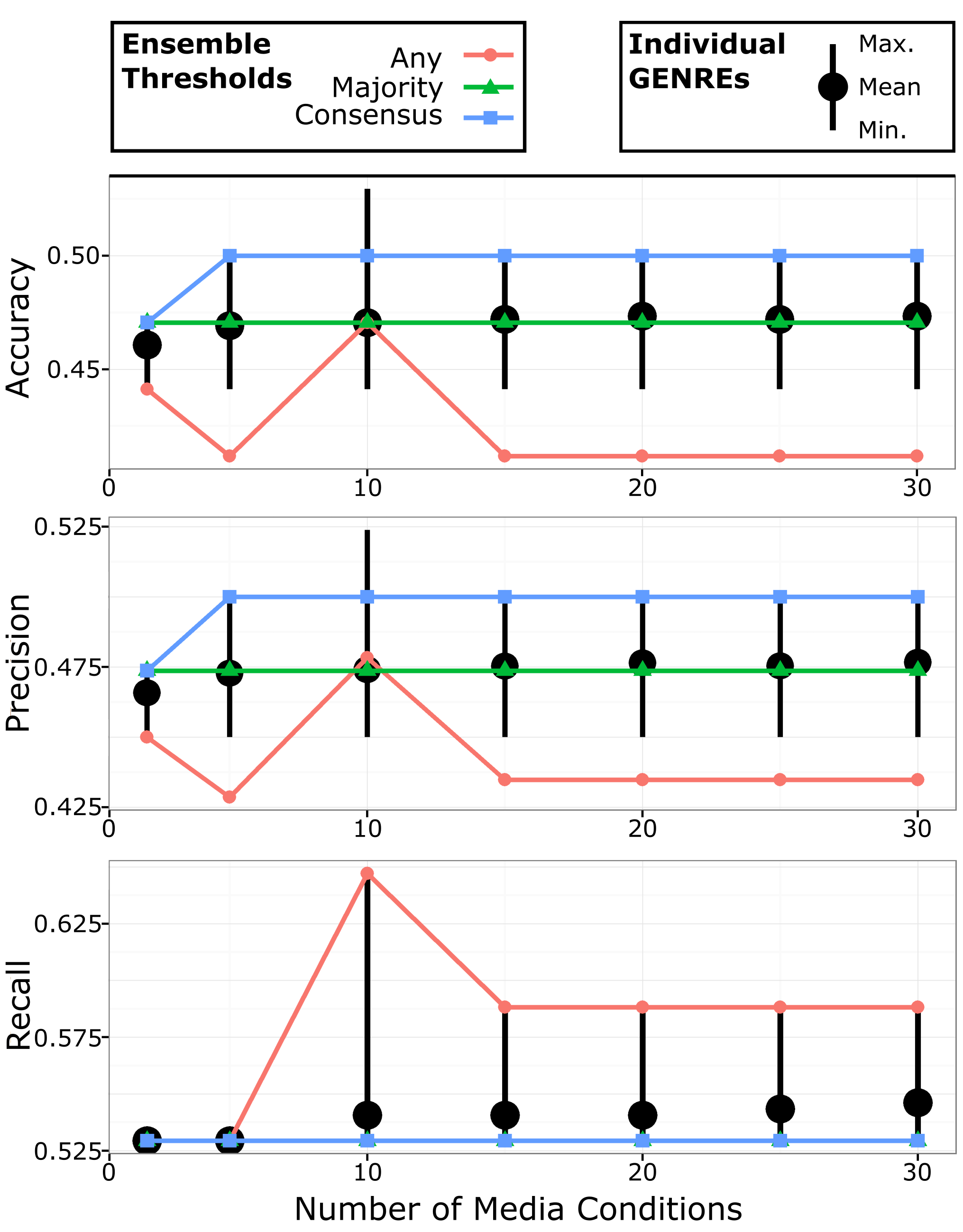

Supplement: S1 Fig — For each number of media conditions, we generated 21 GENREs, where each GENRE was gap filled using a different order of the same input media conditions. Note that for two media conditions only two orders are possible. We evaluated the accuracy, precision, and recall of every individual GENRE and of the ensembles by predicting growth on 17 positive media conditions and 17 negative media conditions which were not used during gap filling. The average of the individual GENREs is shown as black points with the maxima and minima as black lines extended above and below. The ensemble predictions using the three different thresholds are shown as red circles “any”, green triangles “majority”, and blue squares “consensus”. (TIF) [file pcbi.1005413.s001.tif]

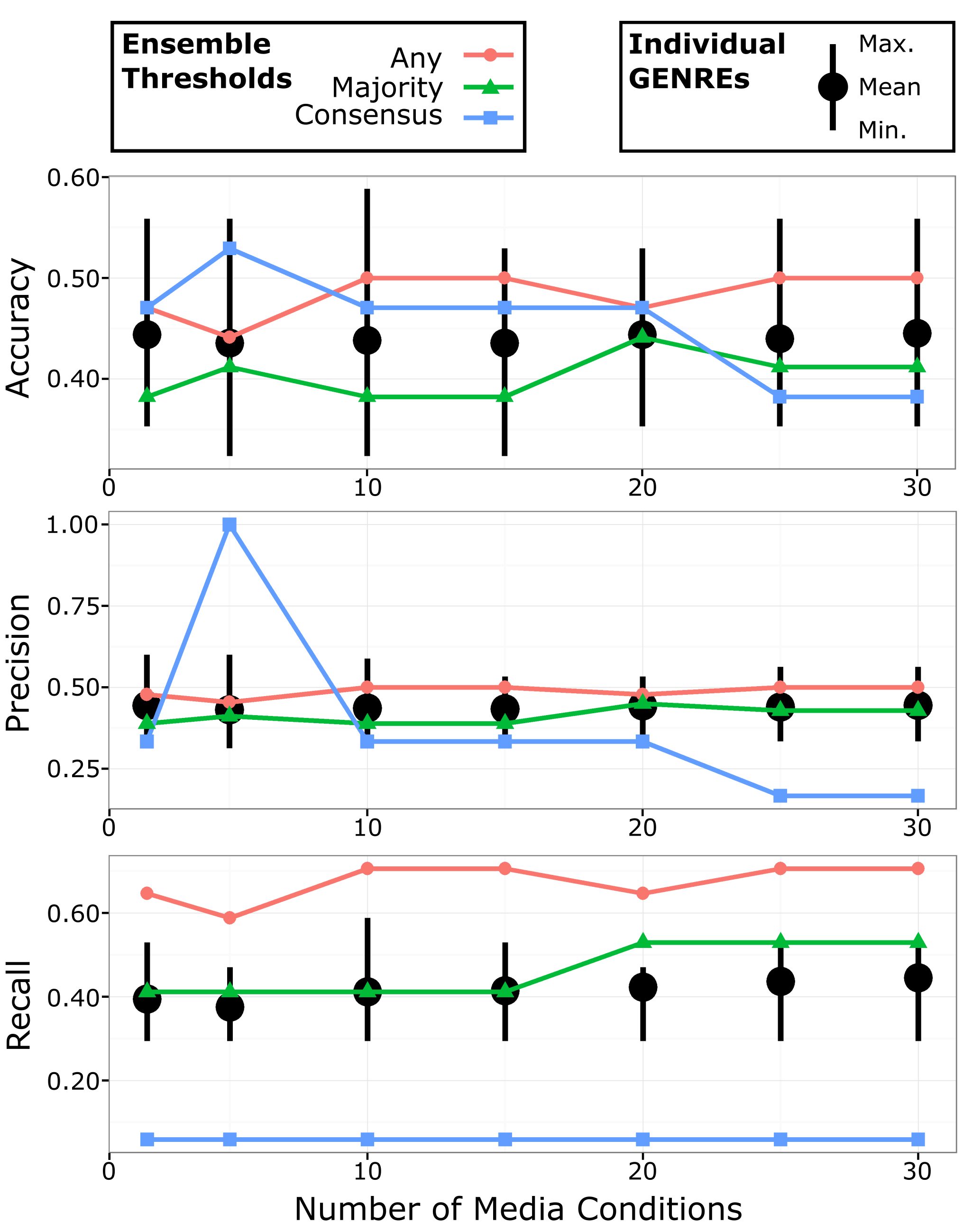

Supplement: S2 Fig — For each number of media conditions, we generated 21 GENREs, where each GENRE was gap filled using a different order of the same input media conditions. Additional diversity was generated by using a stochastic gap fill step and including a random subset of the reactions from the Model SEED draft network. We evaluated the accuracy, precision, and recall of every individual GENRE and of the ensembles by predicting growth on 17 positive media conditions and 17 negative media conditions which were not used during gap filling. The average of the individual GENREs is shown as black points with the maxima and minima as black lines extended above and below. The ensemble predictions using the three different thresholds are shown as red circles “any”, green triangles “majority”, and blue squares “consensus”. (TIF) [file pcbi.1005413.s002.tif]

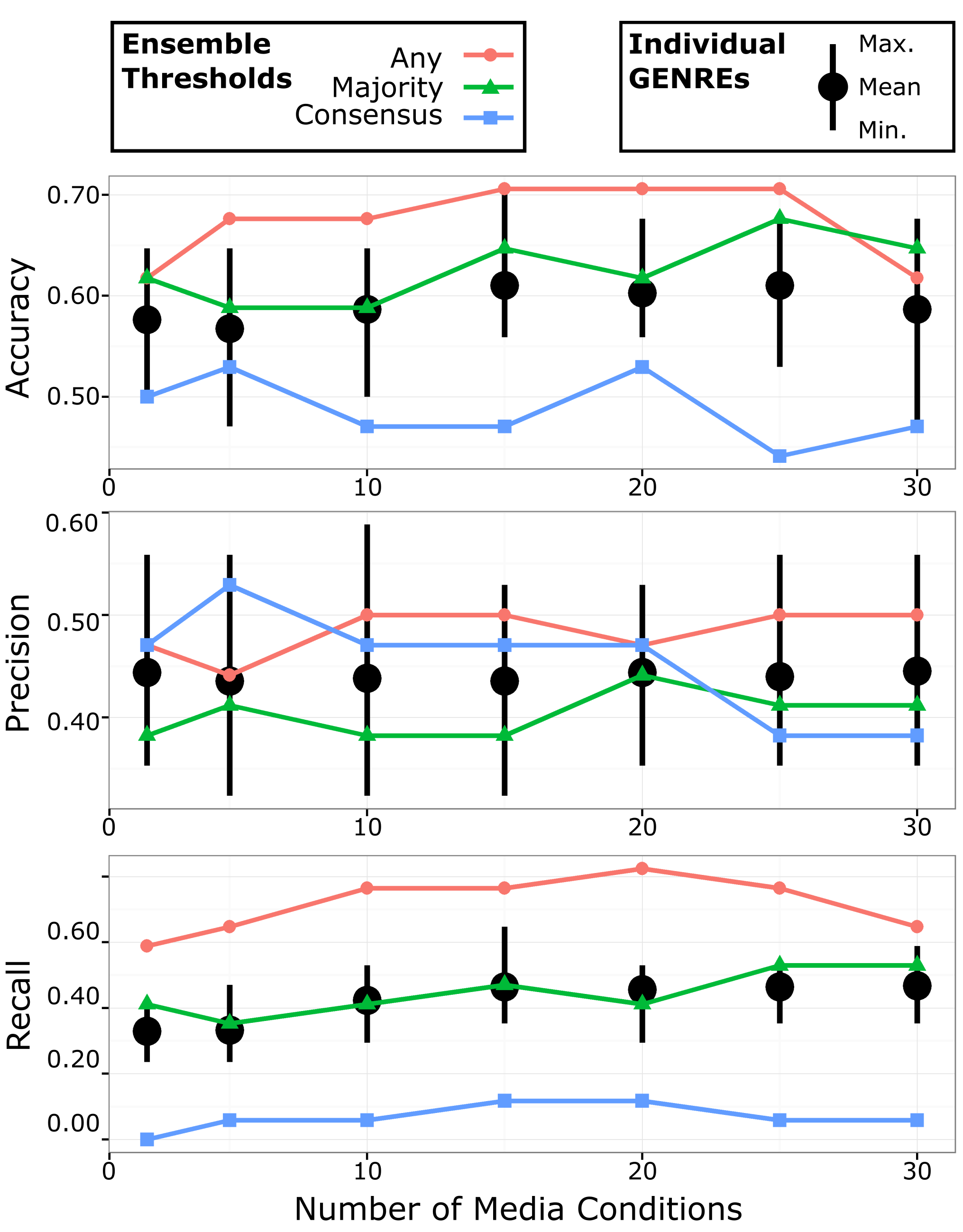

Supplement: S3 Fig — For each number of media conditions, we generated 21 GENREs, where each GENRE was gap filled using a different order of the same input media conditions. Information about 10 randomly-selected negative growth conditions was incorporated through a trimming step. Additional diversity was generated by using a stochastic gap fill step and including a random subset of the reactions from the Model SEED draft network. We evaluated the accuracy, precision, and recall of every individual GENRE and of the ensembles by predicting growth on 17 positive media conditions and 17 negative media conditions which were not used during gap filling. The average of the individual GENREs is shown as black points with the maxima and minima as black lines extended above and below. The ensemble predictions using the three different thresholds are shown as red circles “any”, green triangles “majority”, and blue squares “consensus”. (TIF) [file pcbi.1005413.s003.tif]

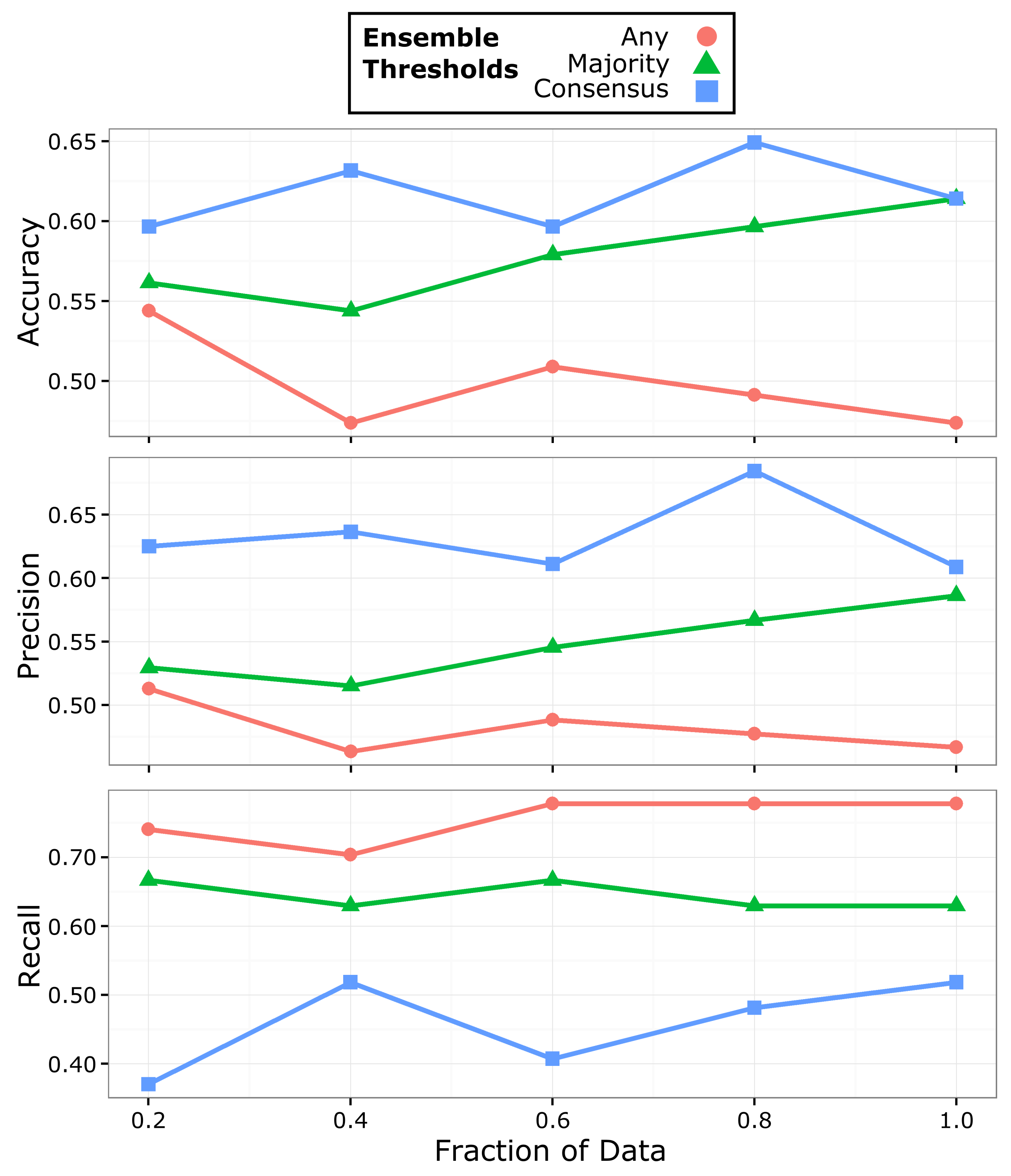

Supplement: S4 Fig — Ensembles were assembled from networks which were trained with increasing fractions of the available data (from 0.2–1.0). For example, to create the individual networks in the ensemble at 0.2, we randomly selected 20% of the growth conditions and 20% of the non-growth conditions. Each individual network was gap filled on a new 20% subset of the data. The individual networks at 1.0 were all gap filled with all the data. The effects of gap filling with subsets of the data are threshold-dependent. We observed that for the “majority” threshold, increasing the fraction of data used to train individual networks increased accuracy and precision while slightly decreasing recall. For the “consensus” threshold, increasing the fraction of data had very little impact on accuracy or precision, but did increase recall. For the “any” threshold, increasing the fraction of data decreased accuracy and precision while improving recall. (TIF) [file pcbi.1005413.s004.tif]
